# Supplementary figures and images for: Impact, obstacles and boundaries of patient partnership: A qualitative interventional study in Lebanon
Source: PLoS One. 2022 Jul 7;17(7):e0270654. doi: 10.1371/journal.pone.0270654 (PMC9262200; doi:10.1371/journal.pone.0270654)

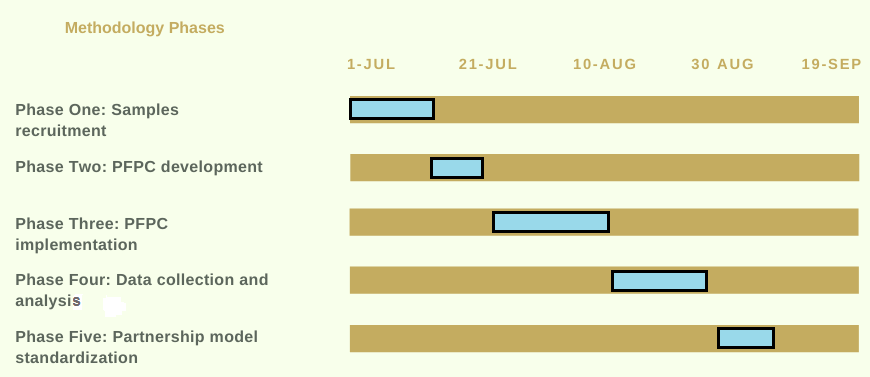

Supplement: S1 Fig — (TIF) [file pone.0270654.s001.tif]
