# Supplementary material for: Impact, obstacles and boundaries of patient partnership: A qualitative interventional study in Lebanon
Source: PLoS One. 2022 Jul 7;17(7):e0270654. doi: 10.1371/journal.pone.0270654 (PMC9262200; doi:10.1371/journal.pone.0270654)
Supplement: S1 File — (DOCX) [file pone.0270654.s002.docx]

**Impact, obstacles and boundaries of patient partnership: a qualitative interventional study in Lebanon**

**Interview Guides**

1. **Qualitative questionnaire for patient partners**

**Team dynamics**

1. How at ease did you feel in joining the patient and family partnership committee PFPC?
2. What was your perception of the welcome by the team (respect, language, fairness, and understanding)?
3. Have you felt a full member of the team?
4. Were you comfortable communicating and enforcing your point of view / your opinions?
5. To what extent did you feel that your views / opinions were taken into account by other members of the PFPC?
6. What has helped your participation in in the PFPC the most?
7. What are the greatest impacts on patients (end users) that will result from the quality improvement recommendation resulted by the PFPC?
8. At what degree do these suggested solutions meet the needs of patients (users)?
9. In your opinion, what did you bring to the team / to the PFPC?

**Learning from experience**

1. What personal benefits (gains) have you derived from your participation in the PFPC?
2. What skills have you acquired during this experience?
3. What are the advantages of contributing to “Partnership Care Program” activities at the healthcare facility?

**Participant satisfaction**

1. What is your overall satisfaction with your involvement in the PFPC?

**Patient partner coordinator**

1. Have you received support from a patient partner–coordinator? If so, what was his/her contribution?

**Challenges**

1. What are the greatest challenges/obstacles related to the implementation of patient partnership activities/the engagement of patient and family partners in the PFPC?

**Opportunities for improvement**

1. What are the recommendations for improvement for future participation in the PFPC?

**Patient partner autonomy boundaries**

1. In your opinion what would you choose when comparing your personal interest against the safety and quality concepts when engaging in the patient care design and delivery decision making?
2. Patient/family personal interest or protecting the common good
3. Patient/family personal interest or applying evidence based practices
4. Patient/family personal interest or protecting people from harm
5. Patient/family personal interest or sense of responsibility to one’s self and others
6. Patient/family personal interest or the medical suggestion
7. Patient/family personal interest or allocating limited resources
8. Patient/family personal interest or healthcare facility rules and regulations
9. Patient/family personal interest or quality and safety experts opinion
10. Patient/family personal interest or nurses’ opinion and recommendations
11. **Qualitative questionnaire for healthcare professionals**

**Team dynamics**

1. What was your perception of the patient and family participation as members in the PFPC?
2. Have you felt them as a full member of the team?
3. Were you comfortable communicating and sharing your opinions with the patient partners?
4. Have you felt in equal position as that of the patient partner in the PFPC?
5. Did you change your language style while communicating with patient partner in the PFPC?
6. Have you felt influenced by the patient partners’ opinion when they have suggested their initiatives?
7. What were the terms of support provided for the patient partners in the committee?
8. In your opinion what resources can provide easy access and participation of patient in the PFPC?

**Challenges**

1. What are the greatest challenges/obstacles related to the implementation of patient partnership activities/the engagement of patient and family partners in the PFPC?

**Opportunities for improvement**

1. What are the recommendations for improvement for future participation in the PFPC?

**Learning from experience**

1. What are the greatest impacts on patients (users) that will result from the quality improvement recommendation resulted by the PFPC?
2. In your opinion, at what degree do the patient partners’ suggested solutions meet the needs of patients (users)?
3. What personal benefits (gains) have you derived from your participation in the PFPC?
4. What skills have you acquired during this experience?
5. What are the advantages of contributing to “Partnership Care Program” activities at the healthcare facility?

**Patient partner coordinator**

1. Have you received support from a patient partner coordinator? If so, what was his/her contribution?

**Participant satisfaction**

1. What is your overall satisfaction with your involvement in the PFPC?
2. Have you felt a change in the quality improvement process and suggested ideas when comparing the traditional process and ideas against when patient and family are engaged?
3. Would you standardize the practice of patient partnership in quality committee?

**Patient partner autonomy boundaries**

1. In your opinion what would you choose when comparing the patient and family personal interest against the ‎safety and quality concepts when engaging the patient in care design and delivery ‎decision making? ‎
2. Patient/family personal interest or protecting the common good
3. Patient/family personal interest or applying evidence based practices
4. Patient/family personal interest or protecting people from harm
5. Patient/family personal interest or resource utilization
6. Patient/family personal interest or sense of responsibility to one’s self and others
7. Patient/family personal interest or the medical suggestion
8. Patient/family personal interest or allocating limited resources
9. Patient/family personal interest or the healthcare facility rules and regulations
10. Patient/family personal interest or quality and safety experts opinion
11. Patient/family personal interest or the nurses’ opinion and recommendations
12. **إستمارة نوعيّة للمرضى والعائلة أو من يمثلهم المشاركين بمشروع الشراكة مع المريض**

**عمل الفريق بلجنة الشراكة مع المريض**

1. هل كان من السهل الإنضمام للجنة المشاركة مع المريض؟

………………………………………………………………………………………………………………………………………………………

1. ما هو إنطباعك عن ترحيب الفريق بك (من جهة الإحترام, اللغة المعتمدة, المساواة, وفهمك للأمور)؟

……………………………………………………………………………………………………………………………………………………….

1. هل شعرت أنك فرد فعّال في الفريق في اللجنة؟

………………………………………………………………………………………………………………………………………………………

1. هل كنت تشعر بالراحة عند تواصلك ومشاركتك أفكارك وآرائك مع الفريق في اللجنة؟

………………………………………………………………………………………………………………………………………………………

1. الى أيّ مدى تعتقد أن آراءك قد نالت إهتمام الفريق في اللجنة؟

………………………………………………………………………………………………………………………………………………………

1. ما هي النقاط التي ساهمت في تعزيز مشاركتك في لجنة المشاركة مع المريض؟

………………………………………………………………………………………………………………………………………………………

1. ما هي التأثيرات الإيجابية على متلقي الرعاية (وهو المريض) التي نتجت عن تقديم إقتراحاتك في اللجنة بخصوص تطوير جودة الرعاية؟

………………………………………………………………………………………………………………………………………………………

1. الى أي مدى تعتقد أن هذه الإقتراحات تتماشى مع حاجات ومتطلبات المريض؟

………………………………………………………………………………………………………………………………………………………

1. برأيك, ما الذي أضفته الى الفريق؟

………………………………………………………………………………………………………………………………………………………

المكتسبات والمنفعة الشخصيّة بعد تجربة الشراكة مع المريض

1. ما هي الفوائد الشخصيّة الناتجة عن مشاركتك في مشروع الشراكة مع المريض؟

………………………………………………………………………………………………………………………………………………………

1. ما هي المهارات التي إكتسبتها من خلال تجربتك؟

………………………………………………………………………………………………………………………………………………………

1. ما هي مميزات مشروع الشراكة مع المريض في المستشفى؟

………………………………………………………………………………………………………………………………………………………

رضى المشاركين

1. ما هي **نسبة** رضاك بشكل عام عن مشروع الشراكة مع المريض؟

دور منسقة مشروع الشراكة مع المريض

1. هل تلقيت الدعم من منسق مشروع الشراكة مع المريض؟ إذا نعم, ما كان دوره؟

………………………………………………………………………………………………………………………………………………………

التحديات

1. ما هي العقبات والتحديات المتعلقة بتطبيق مشروع الشراكة مع المريض, وبإنضمام المريض والعائلة للجنة المشاركة مع المريض؟

………………………………………………………………………………………………………………………………………………………

المقترحات للتحسين

1. ماهي المقترحات التي تقدمها من أجل تطوير مشروع الشراكة مع المريض فيما بعد؟

………………………………………………………………………………………………………………………………………………………

حدود الشراكة مع المرضى

1. برأيك أي من المفاهيم أدناه يطغى على الآخر عند المقارنة في الأمور المتعلقة بنسبة إشراك المريض والعائلة بالتخطيط للرّعاية الصحيّة وتقديمها:
2. الحرية الشخصية للمريض والعائلة أم حماية المصلحة العامة
3. الحرية الشخصية للمريض والعائلة أم تطبيق معايير مثبتة علميّاً
4. الحرية الشخصية للمريض والعائلة أم حماية الآخرين من الخطر ‏
5. الحرية الشخصية للمريض والعائلة أم الإحساس بالمسؤولية نحو الذات والآخرين
6. الحرية الشخصية للمريض والعائلة أم إقتراح الطبيب‎ ‎
7. الحرية الشخصية للمريض والعائلة أم الإستهلاك المدروس للموارد المحدودة
8. الحرية الشخصية للمريض والعائلة أم قوانين وأنظمة المنشأة الصحية
9. الحرية الشخصية للمريض والعائلة أم آراء خبراء الجودة والسلامة
10. الحرية الشخصية للمريض والعائلة أم آراء وإقتراحات الممرض
11. **إستمارة نوعيّة للعاملين الصحيّين**

**عمل الفريق الصحي بلجنة الشراكة مع المريض**

1. ما هو إنطباعك عن إنضمام المريض والعائلة للجنة المشاركة مع المريض؟

………………………………………………………………………………………………………………………………………………………

1. هل شعرت أن المريض والعائلة فرد فعّال في الفريق في اللجنة؟

………………………………………………………………………………………………………………………………………………………

1. هل كنت تشعر بالراحة عند تواصلك ومشاركتك أفكارك وآراءك مع المرضى والعائلة المشاركين في اللجنة؟

………………………………………………………………………………………………………………………………………………………

1. هل شعرت بمساواة في الموقع بينك وبين المرضى والعائلة المشاركين في اللجنة؟

………………………………………………………………………………………………………………………………………………………

1. هل غيرت في مفردات الجودة عند تواصلك مع المرضى والعائلة المشاركين في اللجنة؟

………………………………………………………………………………………………………………………………………………………

1. عند تقديم الإقتراحات من قبل المرضى والعائلة المشاركين في اللجنة, هل تأثرت بأي مقترح منها؟

………………………………………………………………………………………………………………………………………………………

1. ما كانت أبرز النقاط الداعمة للشراكة مع المريض في اللجنة؟

………………………………………………………………………………………………………………………………………………………

1. برأيك ما هي الموارد التي تسهل الإنضمام الى الإجتماعات ومشاركة المريض والعائلة؟

………………………………………………………………………………………………………………………………………………………

1. ما هي التأثيرات الإيجابية على متلقي الرعاية (المريض) التي نتجت عن تقديم المرضى والعائلة إقتراحاتهم في اللجنة بخصوص تطوير جودة الرعاية؟

………………………………………………………………………………………………………………………………………………………

1. الى أي مدى تعتقد أن هذه الإقتراحات تتماشى مع حاجات ومتطلبات المريض؟

………………………………………………………………………………………………………………………………………………………

التحديات

1. ما هي العقبات والتحديات المتعلقة بتطبيق مشروع الشراكة مع المريض, وبإنضمام المريض والعائلة للجنة المشاركة مع المريض؟

………………………………………………………………………………………………………………………………………………………

المقترحات للتحسين

1. ماهي المقترحات التي تقدمها من أجل تطوير مشروع الشراكة مع المريض فيما بعد؟

………………………………………………………………………………………………………………………………………………………

دور منسقة مشروع الشراكة مع المريض

1. هل تلقيت الدعم من منسق مشروع الشراكة مع المريض؟ إذا نعم, ما كان دوره؟

………………………………………………………………………………………………………………………………………………………

المكتسبات والمنفعة الشخصيّة بعد تجربة الشراكة مع المريض

1. ما هي الفوائد الشخصيّة الناتجة عن مشاركتك في مشروع الشراكة مع المريض؟

………………………………………………………………………………………………………………………………………………………

1. ما هي المهارات التي إكتسبتها من خلال تجربتك؟

………………………………………………………………………………………………………………………………………………………

1. ما هي مميزات مشروع الشراكة مع المريض في المستشفى؟

………………………………………………………………………………………………………………………………………………………

رضى المشاركين

1. ما هي نسبة رضاك بشكل عام عن مشروع الشراكة مع المريض؟
2. هل شعرت بأي تغيّر في آلية تحسين الجودة والأفكار المقترحة في اللجنة عند المقارنة بالممارسات السابقة التي لا تتضمن المريض والعائلة؟

………………………………………………………………………………………………………………………………………………………

1. هل توافق على إعتماد لجنة الشراكة مع المريض كآلية رسمية لتحقيق السلامة وتحسين الجودة في المستشفى؟

………………………………………………………………………………………………………………………………………………………

حدود الشراكة مع المرضى

1. برأيك أي من المفاهيم أدناه يطغى على الآخر عند المقارنة, في الأمور المتعلقة بنسبة إشراك المريض والعائلة بالتخطيط للرعاية الصحية وتقديمها:
2. الحرية الشخصية للمريض والعائلة أم حماية المصلحة العامة
3. الحرية الشخصية للمريض والعائلة أم تطبيق معايير مثبتة علميّاً
4. الحرية الشخصية للمريض والعائلة أم حماية الآخرين من الخطر
5. الحرية الشخصية للمريض والعائلة أم الإحساس بالمسؤولية نحو الذات والآخرين
6. الحرية الشخصية للمريض والعائلة أم إقتراح الطبيب
7. الحرية الشخصية للمريض والعائلة أم الإستهلاك المدروس للموارد المحدودة
8. الحرية الشخصية للمريض والعائلة أم قوانين وأنظمة المنشأة الصحية
9. الحرية الشخصية للمريض والعائلة أم آراء خبراء الجودة والسلامة
10. الحرية الشخصية للمريض والعائلة أم آراء وإقتراحات الممرض
